# The WAK/WAKL gene family in *Phaseolus vulgaris*: genomic characterization and expression under *Colletotrichum lindemuthianum* infection

**DOI:** 10.1186/s12864-026-12531-2

**Published:** 2026-01-24

**Authors:** Gabriel César Ferreira, Elenildo dos Santos Oliveira, Welison Andrade Pereira

**Affiliations:** Department of Biology, Institute of Natural Sciences, Universidade Federal de Lavras (UFLA), Lavras, MG Brazil

**Keywords:** Genome-wide analysis, PvWAK, PvWAKL, Common bean, RNA-Seq, Transcriptome, Anthracnose

## Abstract

**Background:**

Wall-associated kinases (WAKs) and WAK-like proteins (WAKLs) are receptor-like kinases implicated in plant perception of extracellular cues and immune signaling. In common bean (*Phaseolus vulgaris*), differential resistance to *Colletotrichum lindemuthianum* offers an opportunity to link structural variation in these receptors to functional outcomes.

**Results:**

Here, we systematically characterize the *WAK/WAKL* gene family in *P. vulgaris*, integrating domain architecture, gene structure, phylogeny, synteny, and transcriptomic responses in resistant (Ouro Vermelho) versus susceptible (BRS Estilo) cultivars across infection time points. Using conserved domain annotation, PvWAKs and PvWAKLs were classified based on combinations of extracellular domains (EGF and GUB_WAK_bind) while retaining a conserved intracellular kinase core. Twenty-five distinct domains were identified, with specific sets being common or exclusive to each group, suggesting modular diversification of interaction interfaces. Phylogenetic and gene structure analyses revealed evolutionary patterns consistent with both conservation of signaling machinery and divergence in extracellular features. Integration with synteny supported retention of candidate orthologs across related species. RNA-Seq profiling at 0, 48, and 96 h post-inoculation uncovered complex, genotype- and time-dependent expression dynamics: subsets of *PvWAK/WAKL* genes were differentially expressed uniquely in resistant or susceptible backgrounds, while others displayed opposing temporal patterns, highlighting nuanced regulatory contributions to resistance. Combining structural and expression evidence yielded a prioritized list of *PvWAK/WAKL* candidates potentially underlying anthracnose resistance.

**Conclusions:**

The study delineates how conserved kinase signaling is coupled to extracellular domain variability and dynamic expression to enable functional divergence within the *WAK/WAKL* family in *P. vulgaris*. The resulting candidate genes provide a focused basis for functional validation and may inform molecular breeding strategies for enhanced disease resistance.

**Supplementary Information:**

The online version contains supplementary material available at 10.1186/s12864-026-12531-2.

## Introduction

The cultivation of common bean (*Phaseolus vulgaris*) holds a prominent position in global agriculture [1], especially in countries where this legume serves as one of the main sources of protein and other essential nutrients, such as complex carbohydrates, fiber, and mineral [2, 3]. In addition to its nutritional relevance, common bean plays a significant economic and social role [1, 2]. However, as with other crops, its productivity is limited by several factors, including adverse edaphoclimatic conditions, soil mineral deficiencies, and both biotic and abiotic stresses [2]. Among the various constraints, anthracnose, caused by the hemibiotrophic fungus *Colletotrichum lindemuthianum*, stands out for its destructive potential, capable of causing total crop loss under favorable conditions for the pathogen, particularly in subtropical and temperate regions [4]. Genetic breeding aimed at improving productivity has shown positive effects on anthracnose resistance [5], supporting the relationship between genetic resistance and agronomic performance.

Research has shown that plants respond to environmental challenges, phytohormones, nutrients, and different types of stress through a complex signaling network [6]. As sessile organisms, plants have developed highly efficient biochemical systems to detect and respond to various extracellular stimuli, with receptor-like protein kinases located in the plasma membrane or associated with the cell wall, playing a central role [7, 8]. The proteomic repertoire involved in these responses is diverse and includes important gene families such as Leucine-Rich Repeat Receptor-Like Kinases (LRR-RLKs) [9, 10], wall-associated kinases (WAKs), and WAK-like kinases (WAKLs) [11–14]. WAKs/WAKLs are notable not only for their role in responding to environmental signals but also for their involvement in key developmental processes, such as cell elongation and reproduction [15, 16].

Structurally, WAKs typically feature a signal peptide, an extracellular domain (ECD) with epidermal growth factor (EGF)-like repeats, a transmembrane region (TM), and an intracellular kinase domain (PK) [17]. The ECD is responsible for recognizing cell wall ligands such as pectins and oligogalacturonides (OGs), triggering intracellular signaling cascades [18, 19]. The transmembrane region anchors these proteins to the plasma membrane, facilitating communication between the extracellular environment and the cytoplasm [12, 20]. In the cytoplasmic space, the kinase domain catalyzes phosphorylation reactions, regulating the transduction of received signals [11, 21]. The physiological functions attributed to WAKs/WAKLs are diverse, and variations in their extracellular domain composition [17] may contribute to functional specificity. These proteins are involved in processes such as cell elongation [22], vegetative development [7, 15, 16, 23], and responses to stresses including salinity [8], cold [13], heat [14, 24], and heavy metals [25, 26]. Their role in phytohormone signaling [19, 27, 28] and especially in pathogen infection responses [29–31] is also noteworthy.

Despite the growing body of knowledge about WAKs/WAKLs and their functions in various species [19, 32–34], information regarding the functional potential of these proteins in *Phaseolus vulgaris* remains scarce. Given the strategic importance of common bean for food security and the various challenges faced by this crop [2], it is essential to identify and characterize this gene family in order to explore its biological potential. Therefore, we identified and characterized the *WAK/WAKL* family in *Phaseolus vulgaris*, evaluating their expression profiles in contrasting cultivars (resistant and susceptible to anthracnose) after inoculation with *Colletotrichum lindemuthianum*. To this end, we analyzed transcriptomes obtained at time points corresponding to the biotrophic and necrotrophic phases of the disease [35]. The results revealed specific candidate genes involved in the immune response of common bean to the pathogen.

## Materials and methods

### Retrieval and identification of PvWAK/WAKL proteins

To identify members of the *WAK/WAKL* gene family in common bean (*Phaseolus vulgaris*), we used the v2.1 genome version available in Phytozome v.13 [36]. The amino acid sequences were analyzed using HMMER v3.2.2 [37], with an E-value threshold < 0.1, based on Hidden Markov Models (HMMs) retrieved from InterPro (https://www.ebi.ac.uk/interpro/). The following HMM profiles were employed: PF00069 and PF07714 (kinase domains); PF13947, PF14380, and PF08488 (GUB_WAK_bind, WAK_assoc, or WAK domains); and PF07645, PF00008, PF12662, and PF12661 (EGF_CA, EGF, cEGF, or hEGF domains). Domain presence was validated using the CDD-NCBI database [38]. Signal peptides and transmembrane regions were predicted using SignalP 6.0 [39] and TMHMM [40], respectively. Proteins were classified as WAKs when they simultaneously contained a kinase domain, WAK domain, EGF domain, transmembrane region, and signal peptide. Proteins possessing a kinase domain and either a WAK or EGF domain, along with a transmembrane region and signal peptide, were classified as WAKLs [41]. For domain confirmation, only matches covering more than 50% of the HMM profile were retained. In cases of transcript variants, only the longest isoform was kept. Proteins not meeting these criteria were excluded.

### Phylogenetic analysis

To investigate the classification and evolutionary relationships of PvWAK/WAKL proteins, three complementary phylogenetic analyses were performed. Multiple sequence alignment was conducted using MAFFT v7.505 [42] with automatic parameter adjustment. All phylogenetic trees were inferred with FastTree v2.1.11 [43] under the JTT + CAT substitution model with 1,000 bootstrap replicates and visualized using the iTOL platform [44].

The first analysis included WAK/WAKL proteins from *Phaseolus vulgaris*, as well as from *Arabidopsis thaliana*, *Nicotiana benthamiana*, *Solanum lycopersicum* [31], and *Hordeum vulgare* [34]. This analysis provided the interspecific framework used to assign clades based on previously established classifications for these species [31, 34].

To examine evolutionary relationships within *P. vulgaris*, a second tree was generated using only the 7 PvWAK and 42 PvWAKL proteins. Six HvWAKL proteins from *H. vulgare* (clade V from the first analysis) were retained as external reference sequences to maintain clade structure and facilitate comparative interpretation.

A third phylogenetic analysis integrated the 49 PvWAK/WAKL proteins with a curated set of 58 experimentally characterized WAK/WAKL homologs from 23 plant species, including monocots, dicots, and crops with known WAK/WAKL-mediated phenotypes (e.g., pathogen resistance, abiotic stress responses, development, hormone signaling). This analysis enabled functional contextualization of PvWAK/WAKL proteins relative to well-studied WAK/WAKL members. The resulting tree is presented in Figure S6, and accession numbers and functional annotations are shown in Table S17.

### Biochemical properties of PvWAK/WAKL and HvWAKL proteins

Biochemical properties of PvWAK/WAKL and HvWAKL proteins were predicted based on amino acid sequences. Molecular weight (MW), isoelectric point (pI), and grand average of hydropathicity (GRAVY) were calculated using tools available on the SMS2 platform (https://www.bioinformatics.org/sms2/) [45]. Signal peptides, transmembrane domains, and topological features were assessed using different bioinformatic tools, including TMHMM [40], CCTOP [46], TOPCONS [47], PROTTER [48], and SignalP 6.0 [39]. The use of multiple tools increased the reliability of signal peptide and transmembrane domain predictions. When two or more tools produced concordant results, the consensus was considered as the final annotation.

### Protein domains, conserved motifs, and subcellular localization

The presence and number of domains in the WAK/WAKL proteins of this study were analyzed using the Conserved Domain Database (CDD) [38], with default settings and an E-value threshold of 0.01 (http://www.ncbi.nlm.nih.gov/Structure/cdd/wrpsb.cgi). Conserved structural motifs were identified using the MEME Suite v5.5.7 [49], configured to search for 20 motifs ranging from 6 to 50 amino acid residues, with an E-value threshold of 0.01 (https://meme-suite.org/meme/tools/meme). Subcellular localization was predicted using CELLO v2.5 [50], ProtComp [51], and WolfPsort [52]. Predictions from all three tools were integrated into a heatmap highlighting the most probable localization sites for each protein.

### Gene structure and chromosomal localization

The exon-intron structures of *WAK/WAKL* genes were analyzed using the corresponding coding sequences (CDS) and transcript data, with detailed visualization performed through the Gene Structure Display Server (GSDS) [53] (http://gsds.cbi.pku.edu.cn/). Chromosomal localization of *PvWAK/WAKL* genes was based on their physical positions retrieved from the Phytozome database, and visualized using PhenoGram [54] (http://visualization.ritchielab.org/phenograms/plot).

Gene clusters were identified based on the physical proximity of *WAK/WAKL* genes along each chromosome. In this study, clusters were defined as groups of two or more genes located consecutively on the same chromosome, without intervening unrelated genes, and positioned within a few thousand base pairs of each other, following the criterion proposed by [17].

### Cis-regulatory elements

Promoter regions comprising 1,500 nucleotides upstream of the transcription start site of *PvWAK/WAKL* and *HvWAKL* genes were retrieved from the database and analyzed to identify cis-regulatory elements (CREs). The sequences were submitted to the PlantCARE database [55] (http://bioinformatics.psb.ugent.be/webtools/plantcare/html/), and the results were thoroughly examined. CREs were classified into eight major categories: Abiotic stress-responsive, Biotic stress-responsive, Core, Development-related, Hormone-responsive, Light-responsive, Unknown, and Unnamed. Classification was primarily based on PlantCARE annotations and complemented with information from the literature. Quantitative analyses were conducted to evaluate the distribution of CREs across genes and clades. Descriptive statistics, including minimum, maximum, and average numbers of CREs per gene and per clade, were calculated. These results provided insights into cis-regulatory patterns, supporting inferences about potential regulatory mechanisms and functional adaptations associated with PvWAK/WAKL and HvWAKL proteins.

### Synteny between *Arabidopsis thaliana*, *Phaseolus vulgaris*, and *Glycine max*

To assess genomic region conservation and infer potential functional relationships, synteny analysis was performed between *P. vulgaris* and two reference species: *A. thaliana* and *G. max*. Although *A. thaliana* is evolutionarily more distant and differs in chromosome number (5 vs. 11), it was included due to its relevance as a model organism in functional genomics. In contrast, *G. max* was selected for its closer evolutionary proximity to *P. vulgaris*.

Chromosomal layouts and gene pair information were generated from genomic and GFF3 files using the “One Step MCScanX” and “Text Merge” functions in TBtools [56]. Syntenic relationships were visualized with the “Multiple Synteny Plot” module, highlighting *P. vulgaris* *WAK/WAKL* proteins. This analysis was not intended to assess full-genome collinearity, but rather to identify conserved *WAK/WAKL* protein pairs potentially involved in analogous biological processes across species. Syntenic orthologs in *A. thaliana* and *G. max* were functionally annotated and subjected to GO enrichment analysis using ShinyGO v0.81 [57], enabling their functional classification.

To complement the synteny analysis and provide functional context for selected *PvWAK/WAKL* genes, pairwise BLASTp searches (BLAST + v2.14.1) were performed against a curated set of 58 experimentally characterized WAK/WAKL proteins reported in the literature (Supplementary Table S17). This dataset included representatives from diverse plant species, such as AtWAKL10 and AtWAKL22 (*Arabidopsis thaliana*), BvWAK1 and BvWAK11 (*Beta vulgaris* subsp. *vulgaris*), CaWAKL20 (*Capsicum annuum*), JrWAK12 (*Juglans regia*), NbWAK12 and NbWAK14 (*Nicotiana benthamiana*), SlWAKL6 (*Solanum lycopersicum*), TaWAKL4 (*Triticum aestivum*), and ZmWAK52 and ZmWAK-RLK1 (*Zea mays*), among others. Default BLASTp parameters were used, and percent identity, E-values, and query coverage were recorded.

### Gene expression analysis (RNA-Seq)

Two contrasting *Phaseolus vulgaris* cultivars, BRS Estilo (susceptible) and Ouro Vermelho (resistant), were selected based on their differential response to *Colletotrichum lindemuthianum* race 65 (isolate Lv134) [4]. Seeds were germinated in trays with Plantmax substrate under controlled greenhouse conditions (24 °C, 95% RH). After the development of primary leaves, plants were inoculated with a 1.2 × 10⁶ conidia/mL suspension prepared from fungal sporulation on bean pods over water agar. Leaves and stems were sprayed and incubated in a moist chamber (100% RH, 25 ± 2 °C) for 48 h [58]. Leaf samples were collected at 0, 48, and 96 h post-inoculation (hpi), and symptoms were assessed nine days later using a 1-to-9 scale. Plants with scores ≤ 3 were considered resistant; those > 3, susceptible [59].

Total RNA was extracted from 12 samples (2 cultivars × 3 time points × 2 replicates) using RNATRIzol^®^, with TURBO^TM^ DNase digestion. RNA integrity was evaluated using a TapeStation 4200 (Agilent), and concentration was measured with a Qubit fluorometer. Libraries were prepared with the TruSeq Stranded Total RNA kit (Illumina) after mRNA enrichment by poly(A) selection and sequenced on an Illumina NextSeq 500 platform (2 × 75 bp, paired-end).

Raw reads were evaluated with FastQC and processed with Trimmomatic to remove adapters and low-quality reads (quality < Q15, length < 32 bp) [60, 61]. Filtered reads were aligned to the *P. vulgaris* v2.1 reference genome (Phytozome v13) using STAR with default parameters of paired-end alignment [62, 63]. Gene-level read counts were generated with featureCounts, and normalization and differential expression analyses were performed using DESeq2 (v1.38.0) in R (v4.2). Genes with an adjusted p-value (FDR) < 0.05 and an absolute log₂ fold change ≥ 1.5 were considered differentially expressed (DEGs). For biological interpretation, DEGs associated with resistance were defined as genes up-regulated in the resistant cultivar (Ouro Vermelho) and/or down-regulated in the susceptible cultivar (BRS Estilo). Conversely, DEGs associated with susceptibility were defined as genes down-regulated in the resistant cultivar and/or up-regulated in the susceptible one. Transcriptome comparisons were performed across six contrasts: OV^48hpi^ x E^48hpi^; OV^48hpi^ x OV^0hpi^; E^48hpi^ x E^0hpi^; OV^96hpi^ x E^96hpi^; OV^96hpi^ x OV^48hpi^; E^96hpi^ x E^48hpi^. Comparisons between 0 and 96 hpi were not included because the contrast merges distinct infection phases (biotrophic vs. necrotrophic), increasing biological dispersion and reducing interpretability in a phase-focused analysis. RNA-Seq data used in this study were previously generated and have been deposited in the NCBI GEO repository under accession PRJNA1219552 ([35].

### Manuscript preparation

Text refinement was assisted by a large language model (ChatGPT). All final decisions regarding content, interpretation, and presentation were made by the authors.

## Results

### Retrieval and identification of PvWAK/WAKL proteins

To identify WAK/WAKL proteins in *Phaseolus vulgaris*, we screened the proteome using HMMER and Hidden Markov Models (HMMs) targeting characteristic domains (Table Supplementary 1–9). A total of 34 proteins contained EGF domains (PF00008, PF07645, PF12661, PF12662), 105 had WAK domains (PF08488, PF13947, PF14380), and 1,808 contained kinase domains (PF00069, PF07714). Epidermal growth factor-like (EGF) domains are cysteine-rich extracellular modules associated with ligand perception and structural stabilization. Wall-associated kinase (WAK) domains participate in sensing cell-wall-derived signals, while kinase domains mediate downstream intracellular phosphorylation cascades. When we applied a minimum HMM coverage threshold of 50% and retaining only the longest isoform per gene reduced these numbers to 29 EGF-containing proteins, 88 with WAK domains, and 1,213 with kinase domains. The filtered set was further analyzed for combined presence of EGF, WAK, and kinase domains, as well as transmembrane regions (TM) and signal peptides (SP), using CDD-NCBI, TMHMM, and SignalP 6.0. Based on these criteria, we identified a final set of 7 PvWAKs and 42 PvWAKLs (Table S1-S9). In this study, WAKs are defined as receptor-like kinases containing a signal peptide, GUB_WAK_bind and/or EGF domains, a transmembrane region, and an intracellular serine/threonine kinase domain, whereas WAK-like kinases (WAKL) share the same overall architecture but may lack one or more of the extracellular WAK-associated domains.

To validate our identification approach, we compared the seven PvWAK proteins identified in this study with the six PvWAK1–6 proteins reported by [64]. Five PvWAKs corresponded directly between the two studies, whereas the protein encoded by the WAK1 gene (*Phvul.001G066200.1*) was not retained because it lacked a predicted signal peptide, as confirmed by SignalP 6.0, Protter, CCTOP, and TOPCONS. Two additional gene loci, *Phvul.003G249300.1* and *Phvul.007G138200.1*, were identified as new PvWAK members (PvWAK04 and PvWAK05). These differences likely reflect distinct criteria for signal peptide detection and domain annotation.

To investigate evolutionary relationships, infer potential functional homology [19], and classify the PvWAK/WAKL into five clades previously described in the literature [17], we constructed a first phylogenetic tree (Fig. S1) using 222 WAK/WAKL proteins, including 49 PvWAK/WAKLs identified in this study. For this analysis, we retrieved 26, 29, and 38 sequences from *Arabidopsis thaliana*, *Solanum lycopersicum*, and *Nicotiana benthamiana*, respectively, as reported by Zhong et al. [31]; and 80 sequences from *Hordeum vulgare* from Tripathi et al. [34]. Before phylogenetic inference, sequences containing undetermined residues (‘X’) were excluded.

Clade I contained the highest number of proteins in the interspecific dataset (117), whereas Clade III comprised the largest number of PvWAK/WAKLs (28), indicating that this group may be particularly expanded in *P. vulgaris*. No PvWAK/WAKL was found in Clade V, which led to the selection of six *H. vulgare* proteins from that clade as external reference sequences in subsequent analyses, to assist in clade delimitation and support phylogenetic interpretation.

### Phylogenetic analysis of *Phaseolus vulgaris* WAK/WAKL proteins

**Fig. 1:**
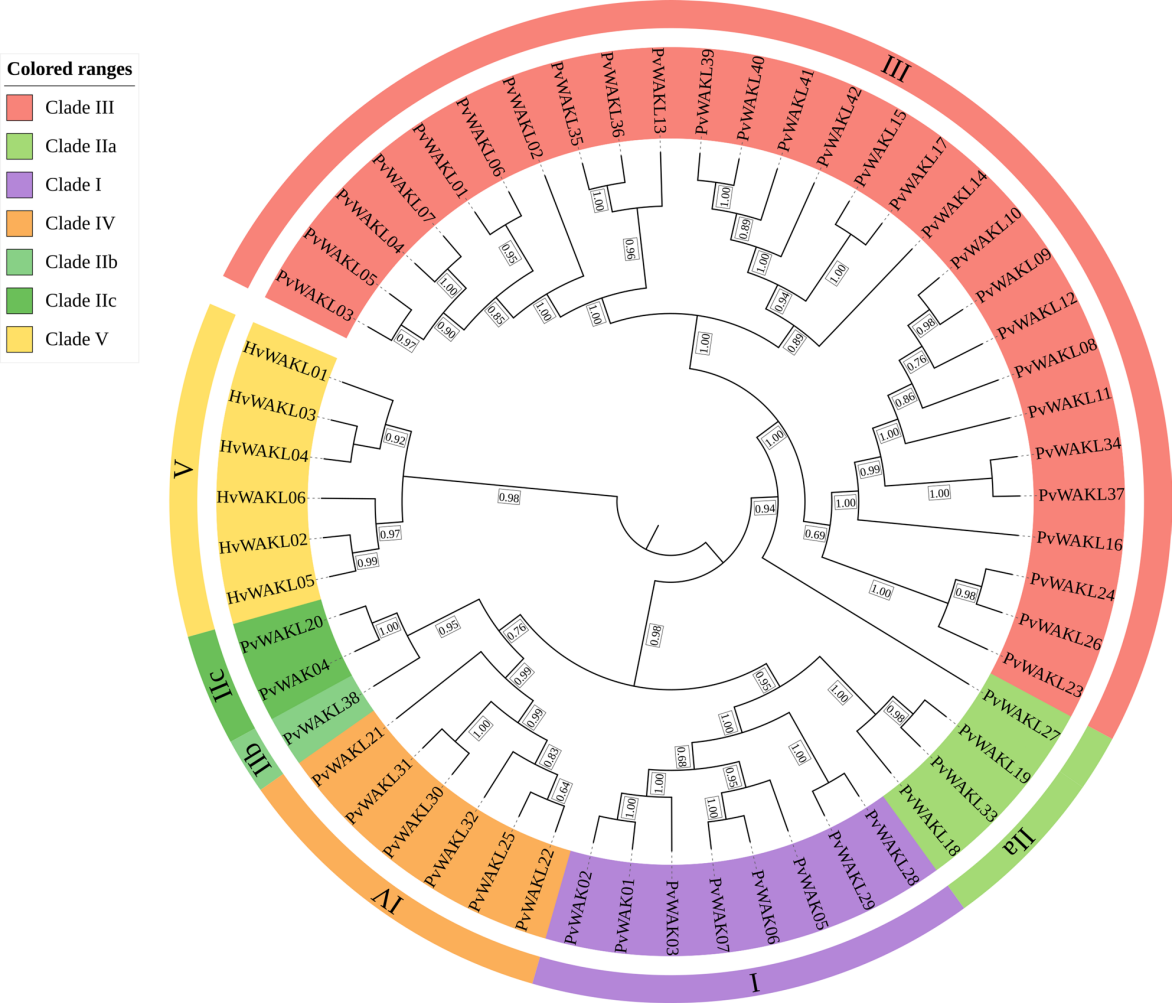
Phylogeny of HvWAKL and PvWAK/WAKL proteins with clade assignments. Maximum-likelihood phylogenetic tree of *Hordeum vulgare* WAKL (HvWAKL) and *Phaseolus vulgaris* WAK/WAKL (PvWAK/WAKL) proteins. Protein sequences were aligned with MAFFT (initial alignment with --auto followed by refinement using --maxiterate 1000 --globalpair), and the tree was inferred with FastTree using the LG model with gamma-distributed rates. Bootstrap support values (from 1,000 replicates) are shown adjacent to branches as numeric labels. Major clades (I–V) are indicated by colored outer arcs (purple: Clade I; light green: Clade IIa; darker green shades: Clades IIb/IIc; salmon/red: Clade III; orange: Clade IV; yellow: Clade V), with Roman numerals marking the clade designations. HvWAKL sequences appear in Clade V, serving as external reference sequences

A second phylogenetic tree was reconstructed using 7 PvWAKs and 42 PvWAKLs from *Phaseolus vulgaris*, along with 6 HvWAKLs from *Hordeum vulgare* as external reference sequences (Fig. 1). In total, 55 proteins were analyzed, which grouped into five major clades. Clade I comprised eight proteins (six PvWAKs and two PvWAKLs), while Clade II was further subdivided into three subclades (IIa, IIb, and IIc). Clade III was the largest, containing 28 PvWAKLs and showing signs of subdivision into IIIa and IIIb. Clade IV included six PvWAKLs, and Clade V consisted exclusively of the six HvWAKLs.

These results suggest that proteins with similar structural characteristics tend to cluster within the same clade, indicating potential evolutionary relationships [65], as observed for PvWAKs in Clade I, PvWAKLs in Clade IV, and HvWAKLs in Clade V. High bootstrap support values further reinforce the robustness of these groupings, providing a solid foundation for future investigations into the evolution and functional divergence of WAK/WAKL proteins in *P. vulgaris*.

### Biochemical, structural, and subcellular features of WAK/WAKL proteins

**Fig. 2:**
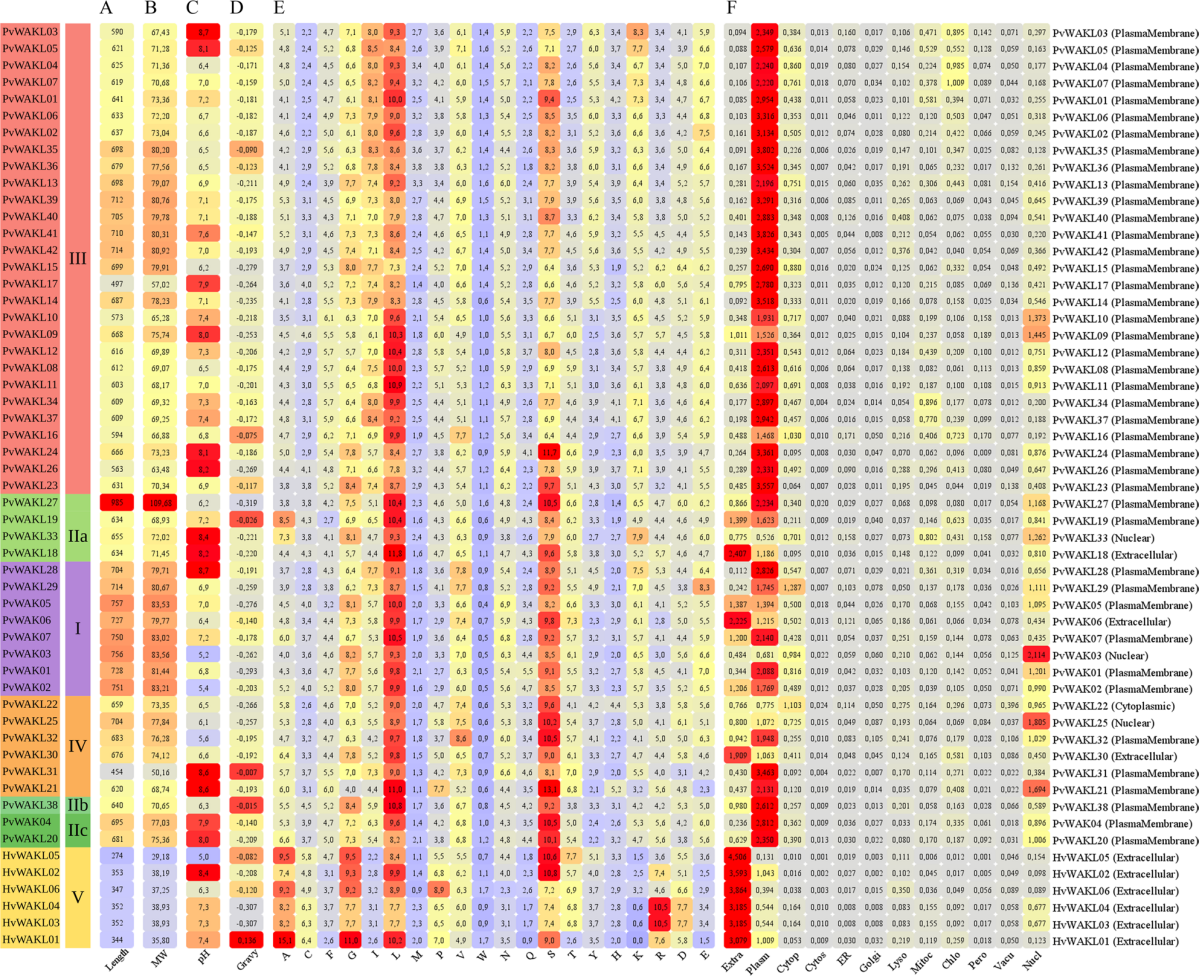
Biochemical properties and localization predictions of HvWAKL and PvWAK/WAKL proteins. Heatmap summarizing structural and biochemical features and predicted subcellular localization for *Phaseolus vulgaris* WAK/WAKL (PvWAK/WAKL) and *Hordeum vulgare* WAKL (HvWAKL) proteins. Proteins are grouped by clade (I–V, indicated by the colored bar at left; color scheme consistent with Fig. 1). Columns represent: (**A**) sequence length (amino acids), (**B**) molecular weight (kDa), (**C**) isoelectric point (pI), (**D**) GRAVY, grand average of hydropathy, (**E**) amino acid composition as percentage, and (**F**) most likely subcellular localization. Values in A–E are color-coded on a continuous scale (e.g., blue = lower, yellow = intermediate, red = higher)

Primary structure analysis provided insights into the biochemical and structural diversity of PvWAK/WAKL proteins. The average protein length was 628 amino acids, ranging from 274 to 985 (Fig. 2 A), which corresponded to molecular weights between 29.18 and 109.68 kDa (mean: 70.41 kDa) (Fig. 2B). Proteins from Clade I, mostly PvWAKs, exhibited the highest lengths and molecular weights, consistent with the presence of larger extracellular domains. In contrast, HvWAKLs from Clade V showed the lowest values, which can be explained by the complete lack of the kinase domain, transmembrane region, and signal peptide. These contrasts reflect domain composition differences and likely functional divergence. It is important to note, however, that the HvWAKL proteins included in this analysis were not identified according to the criteria established in this study; they were retained solely for comparative purposes, as external references derived from the global phylogenetic tree that included WAK/WAKL proteins from other species to support the classification of PvWAK and PvWAKL members.

As observed in other species, including *Cannabis sativa*, *Gossypium hirsutum*, *Pisum sativum*, *Solanum lycopersicum*, and *Zea mays* [28, 66–68], isoelectric point (pI) values ranged from 5.00 to 8.68 (mean: 7.11), indicating acidic, neutral, and basic proteins (Fig. 2 C). GRAVY scores varied from − 0.319 to 0.136 (mean: −0.091) (Fig. 2D), with only HvWAKL01 showing a positive value. Despite the presence of transmembrane domains, 53 of the 54 proteins (98.10%) showed negative GRAVY scores, indicating a predominantly hydrophilic character, consistent with extracellular interaction roles. Negative GRAVY values reflect an overall enrichment in hydrophilic amino acids, whereas positive values indicate greater hydrophobicity. Clades I and V displayed notable distinctions in length, molecular weight, and GRAVY profiles, suggesting structural divergence across clades. Nevertheless, conserved features were also evident. Amino acid composition analysis (Fig. 2E) revealed that leucine (L) was the most abundant amino acid in 36 of the 55 proteins (65.45%), whereas serine (S) predominated in 13 proteins (23.64%). Alanine (A) and arginine (R) were the most abundant residues in two proteins each (3.64%), while isoleucine (I) and glycine (G) predominated in one protein each (1.82%).

Subcellular localization predictions indicated that 27 (55.10%), 42 (85.71%), and 30 (61.22%) of the 49 PvWAK/WAKL proteins were located at the plasma membrane according to ProtComp, CELLO, and WolfPsort, respectively (Fig. 2 F), consistent with their role in transmembrane signaling. In contrast, HvWAKLs from clade V were predicted to be extracellularly localized, which may relate to their high cysteine content, a distinctive feature of this group, and also to the fact that 5 out of 6 proteins lack a transmembrane domain according to TMHMM, and 4 out of 6 according to CCTOP and TOPCONS, although all retain a signal peptide. The combination of these characteristics further supports the predicted extracellular localization and suggests potential roles in the apoplastic environment (Table S10).

### Protein domains and conserved structural motifs

**Fig. 3:**
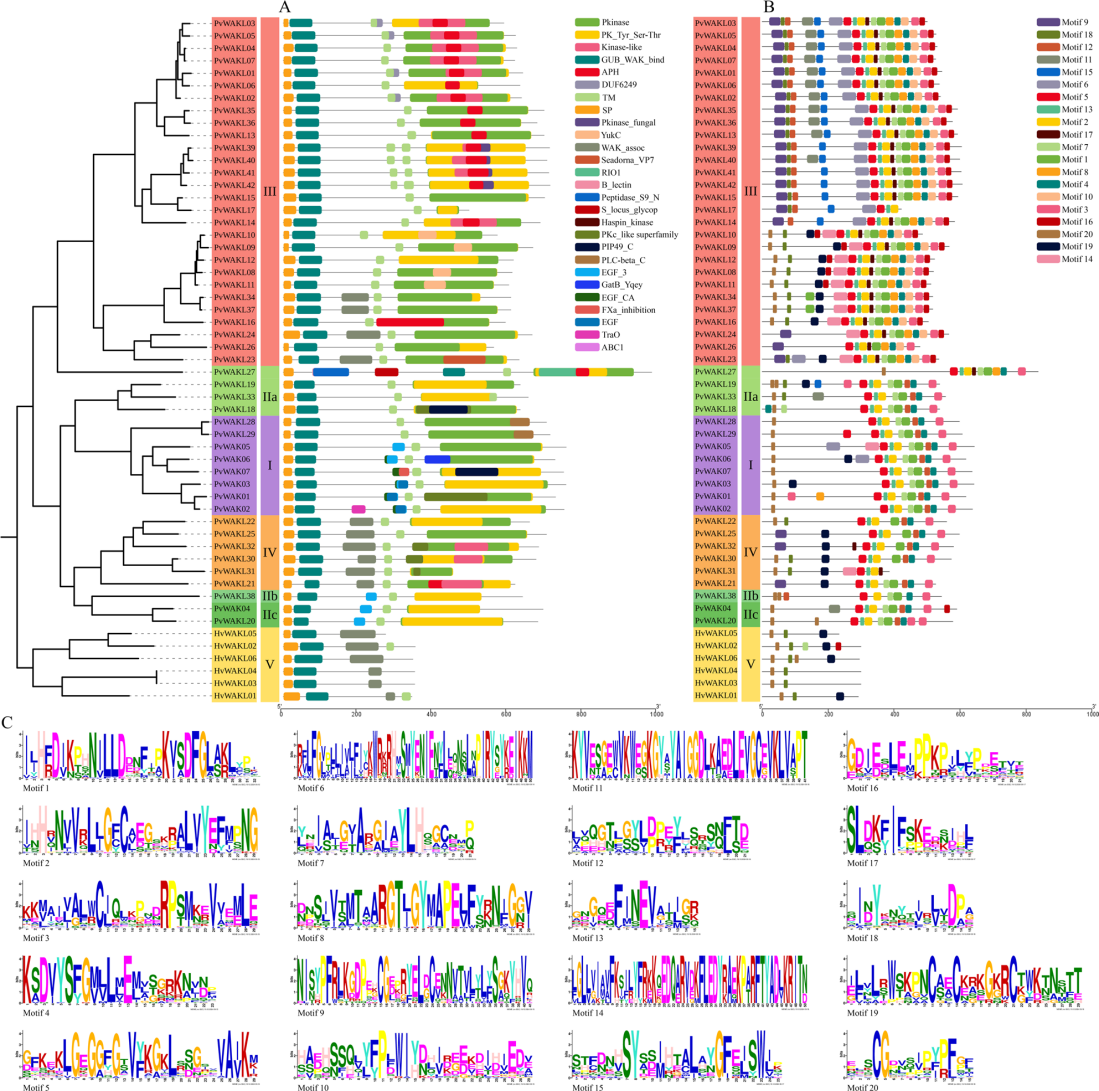
Conserved domains and structural motifs of HvWAKL and PvWAK/WAKL proteins. **A** Conserved protein domains in *Hordeum vulgare* HvWAKL (HvWAKL) and *Phaseolus vulgaris* WAK/WAKL (PvWAK/WAKL) sequences as identified by CDD-NCBI. Domains are color-coded and aligned to each protein in phylogenetic order (clade designations consistent with Figs. 1 and 2), highlighting shared and group-specific architectural features. **B** Structural motifs discovered with MEME Suite across the same protein set; each colored block represents one of the 20 most significant motifs, positioned according to its location within the protein sequence. **C** Sequence logos for the 20 MEME-derived motifs, summarizing residue conservation and positional information for each motif

To structurally characterize the PvWAK/WAKL proteins, domain prediction was performed using CDD-NCBI (Fig. 3 A). All proteins retained the conserved intracellular kinase region, whereas the extracellular portion showed the expected variation that separates WAKs from WAKLs (Fig. S2). Accordingly, PvWAKs consistently displayed a signal peptide, a transmembrane region, kinase, EGF, and GUB_WAK_bind domains [17], whereas proteins lacking one or both extracellular modules (EGF or GUB_WAK_bind) but preserving the kinase domain were classified as PvWAKLs [41]. Notably, 48 of the 49 proteins (97.9%) displayed the GUB_WAK_bind domain near the N-terminus, immediately after the signal peptide and within the first third of the sequence, with PvWAKL27 as the only exception. In addition to these core elements, auxiliary domains (e.g., APH, DUF6249, Pkinase_fungal, WAK_assoc) contributed to differences in protein length and molecular weight (Table S11), indicating potential functional diversification within the family.

In total, 25 distinct domains were predicted, revealing patterns of both conservation and group specificity (Fig. S3). In PvWAKs, the domains common to all members were Pkinase, GUB_WAK_bind, PK_Tyr_Ser-Thr, and EGF_3, whereas FXa_inhibition, TraO, EGF_CA, EGF, and GatB_Yqey were exclusive to this group. In PvWAKLs, GUB_WAK_bind and PK_Tyr_Ser-Thr were shared across all proteins, while PLC-beta_C, S_locus_glycop, Seadorna_VP7, YukC, Pkinase_fungal, DUF6249, APH, B_lectin, Peptidase_S9_N, ABC1, Kinase-like, *and* RIO1 were observed only in this group. These patterns reinforce the idea that, although the internal signaling machinery is preserved, diversity in the extracellular domains may mediate interaction specificity and functional divergence (Table S11).

Structural motifs identified with MEME Suite revealed both conserved and clade-specific patterns (Fig. 3B). Among the 20 predicted motifs, the most frequent were associated with kinase domains (motifs 1 and 5) and GUB_WAK_bind (motif 9). The RDxxxxN pattern, typical of RD-kinases, was found in motif 1, present in all 7 PvWAKs and 11 PvWAKLs (Fig. 3 C), supporting their role in phosphotransfer [7, 30]. WAK/WAKLs contained between 2 and 17 motifs. PvWAKLs from clade III displayed the greatest diversity, while HvWAKLs from clade V exhibited simpler structures, with only five recurrent motifs. Motif 10 was exclusive to clade III, suggesting functional specialization, whereas conserved motifs such as 2, 5, and 13 highlighted residues important across multiple groups (Table S12).

### Gene structure and chromosomal distribution

**Fig. 4:**
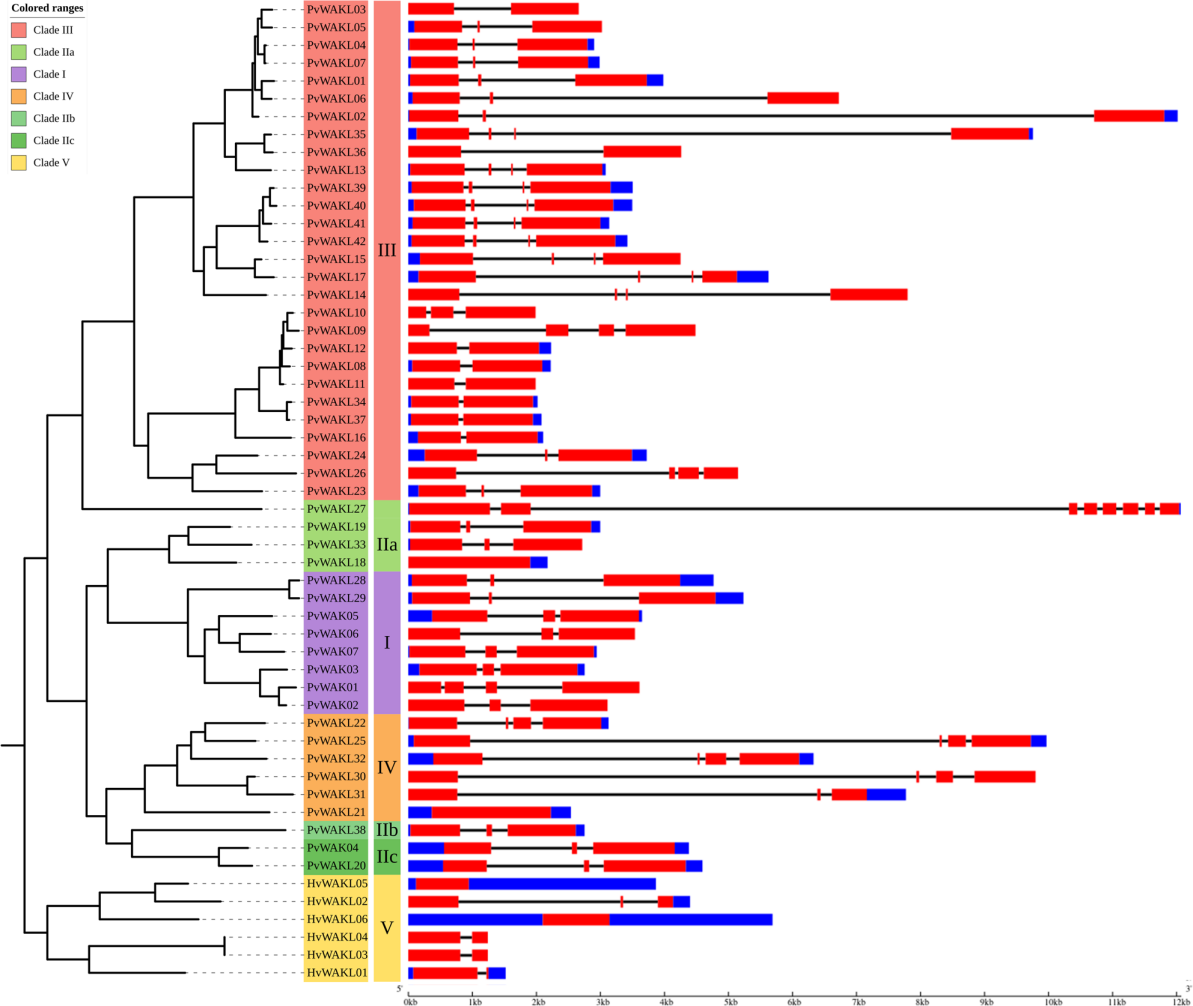
Phylogeny and gene structures of *HvWAKL* and *PvWAK/WAKL* genes. Maximum-likelihood phylogenetic tree (left) of *Hordeum vulgare* WAKL (HvWAKL) and *Phaseolus vulgaris* WAK/WAKL (PvWAK/WAKL) proteins (same inference parameters as in Fig. 1) alongside their corresponding gene structures (right). Gene models are shown in genomic scale (kb), with colored boxes representing structural features (e.g., red = coding exons, blue = UTRs) and connecting lines indicating introns

**Fig. 5:**
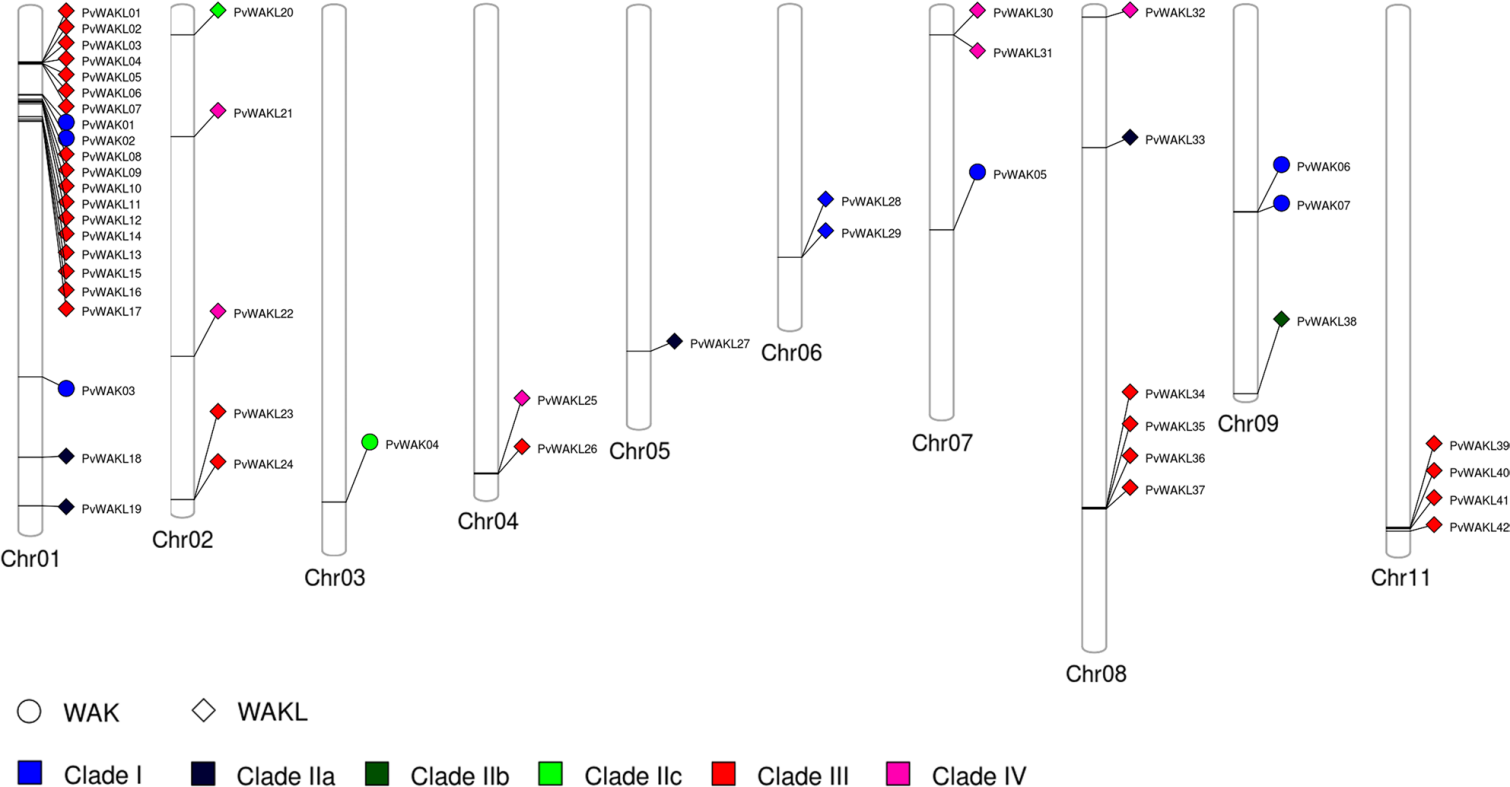
Chromosomal distribution of *PvWAK* and *PvWAKL* genes in *Phaseolus vulgaris*. Genomic positions of *PvWAK* and *PvWAKL* genes mapped onto *Phaseolus vulgaris* *chromosomes*. Each gene is represented by a shape indicating type (circle: *PvWAK*; diamond: *PvWAKL*) and colored according to its clade assignment (blue: Clade I; dark blue/navy: Clade IIa; green: Clade IIb; light green: Clade IIc; red: Clade III; magenta: Clade IV). Chromosome identifiers are shown below each vertical bar, and the relative positions reflect physical coordinates (Mb) extracted from the reference genome

Gene structure analysis revealed that 39 genes in this study harbored two or three introns, consistent with observations in soybean [69]. Four genes (*PvWAKL18*, *PvWAKL21*, *HvWAKL05*, and *HvWAKL06*) lacked introns entirely, while 11 genes had a single intron, and *PvWAKL27* contained seven (Fig. 4 and Table S13). Although *WAK/WAKL* genes typically contain 1–3 introns [7, 11, 30, 70, 71], we observed subtle structural diversity across clades. When gene structure was analyzed alongside chromosomal localization (Fig. 5), consistent intron patterns emerged across the chromosomes, potentially reflecting gene family expansion via tandem or segmental duplication, as reported in previous studies [8, 32, 69]. Genes located on chromosomes 3, 4, 6, and 11 tended to have more introns (3), while those on chromosomes 1, 2, and 9 had fewer (2). *HvWAKL*s contained up to two introns; two genes lacked introns, three contained one intron, and one contained two.

*PvWAK/WAKL* genes were unevenly distributed across 10 of the 11 *P. vulgaris* chromosomes (Fig. 5), a distribution pattern commonly observed in this family across species [67, 69, 72]. The number of genes per chromosome ranged from 1 to 22, with chromosome 1 harboring the largest number. Using our proximity criterion, defined as two or more genes positioned consecutively on the same chromosome and separated by short intergenic distances, we identified nine genomic clusters. The clustered genes were located within 3.1 to 47.7 kb of each other, consistent with the criteria adopted in this study. An additional pair on chromosome 11 (*Phvul.011G198500.1*–*Phvul.011G200400.1*), although physically close in a broader chromosomal context, was separated by ~ 226 kb and therefore not classified as a true cluster. Although two *PvWAKL* genes on chromosome 6 were initially positioned in close proximity, they were separated by an unrelated gene and, therefore, were not classified as part of the same cluster. This clustered organization aligns with observations in other plant species, suggesting potential phylogenetic relatedness and the contribution of tandem duplication to the expansion of the *WAK/WAKL* family [11, 21, 33]. Similar clustered patterns reported in *Solanum lycopersicum* also exhibit conserved motif architecture, reinforcing the functional relevance of such genomic arrangements [7, 70] (Table S13).

### Cis-regulatory elements

A total of 6,370 cis-regulatory elements (CREs), classified into 100 types across six major functional categories, were identified in the promoter regions of *PvWAK/WAKL* genes (Fig. S4). The core promoter elements TATA-box and CAAT-box were the most abundant, occurring 2,092 and 1,439 times, respectively (Fig. S4A). CREs associated with abiotic stress responses (ASR; Fig. S4B) and light responsiveness (LR; Fig. S4G) were also highly represented, with 652 and 548 elements identified, respectively. The number of CRE types varied substantially across categories, ranging from only four in the biotic stress response (BSR) category (Fig. S4C) to 28 in light-responsive (LR) elements, highlighting the need for category-specific interpretation (Table S14).

**Fig. 6:**
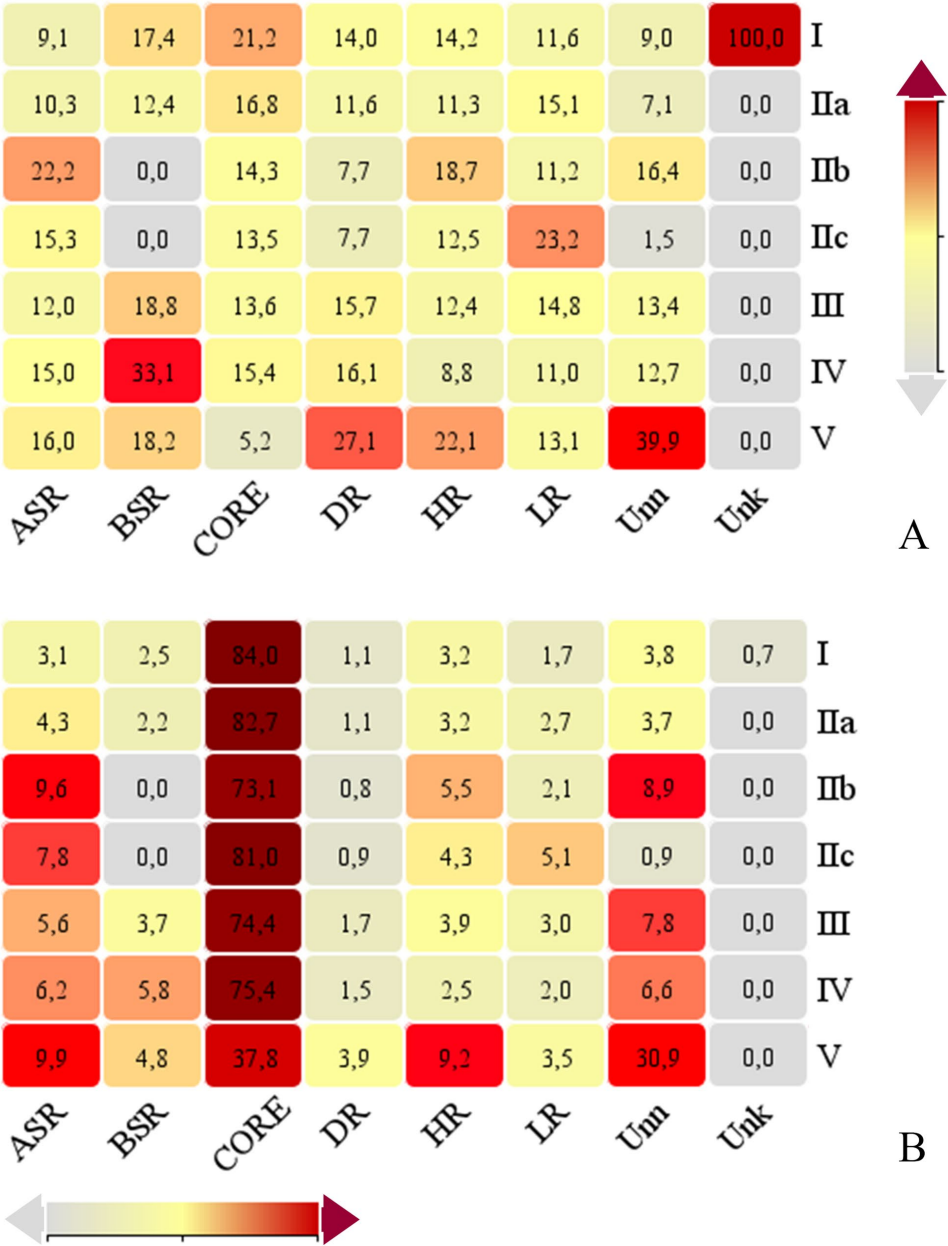
Cis-regulatory element enrichment across PvWAK/WAKL clades and categories. Heatmaps summarizing enrichment patterns of cis-regulatory element categories among *Phaseolus vulgaris* WAK/WAKL (PvWAK/WAKL) clades. In panel **A**, each cell shows the relative proportion or enrichment score of a given category within a clade, enabling vertical comparison of clades for each element type. In panel **B**, each clade’s profile is compared horizontally across regulatory categories, highlighting within-clade variation. Categories on the x-axis are: ASR (Abiotic Stress Responsive), BSR (Biotic Stress Responsive), CORE, DR (Development-related), HR (Hormone Responsive), LR (Light Responsive), Unn (Unnamed), and Unk (Unknown). Color intensity representes enrichment magnitude (scale bar at right: light = lower enrichment; dark red = higher enrichment), with numeric values overlaid in each cell

When analyzing CRE distribution per clade (Fig. 6 A), genes from Clade IIb accounted for 22.2% of all ASR-related CREs, whereas Clade IV genes were prominent in BSR-related elements. Clade IIc showed enrichment in light-responsive (LR) elements, and Clade V exhibited high relative proportions of CREs related to development (DR) and hormone responsiveness (HR).

Comparative analysis across categories within each clade (Fig. 6B) further supported functional divergence. ASR elements were particularly enriched in Clades IIb, IIc, and V. Clade IIc had the highest relative proportion of LR elements, while Clades IV and V showed notable enrichment for BSR and HR elements, respectively. These patterns suggest potential clade-specific regulatory specializations, which may underlie functional divergence in response to environmental cues and developmental signals.

### Synteny among *Arabidopsis thaliana*, *Phaseolus vulgaris*, and *Glycine max*

**Fig. 7:**
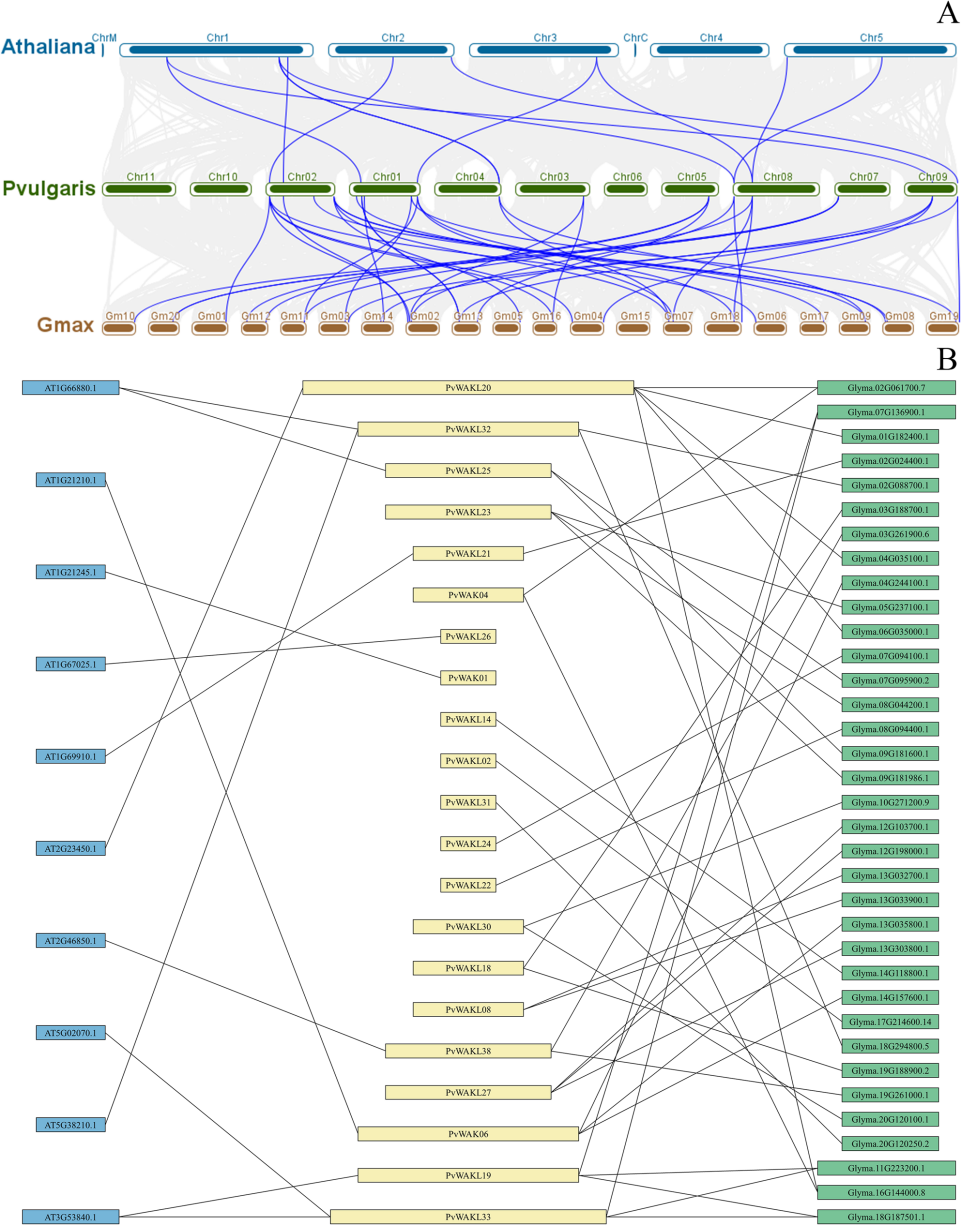
Synteny relationships of WAK/WAKL genes among *Arabidopsis thaliana*, *Phaseolus vulgaris*, and *Glycine max*. **A** Genome-wide synteny map illustrating conserved chromosomal regions among *Arabidopsis thaliana* (top, blue),* Phaseolus vulgaris* (middle, green), and *Glycine max* (bottom, brown). Colored lines represent syntenic connections linking genomic segments that harbor *WAK/WAKL* genes across the three species. **B** Gene-level synteny network decipting homologous relationships among *WAK/WAKL* genes: A. thaliana genes (left, blue), *P. vulgaris WAK/WAKL* (*PvWAK/WAKL*) genes (center, yellow), and *G. max* homologs (right, green). Edges connect genes sharing inferred orthologous or syntenic relationships, highlighting conserved evolutionary links within the *WAK/WAKL* family

The comparative synteny analysis revealed conserved genomic relationships between* P. vulgaris* *WAK/WAKL* genes and their homologs in *G. max* and *A. thaliana* (Fig. 7A). Between the two legumes, 40 conserved collinear relationships were detected, involving 19 *PvWAK/WAKL* genes and their corresponding *GmWAK/WAKL* loci. Six *PvWAK/WAKL*s showed three or more syntenic correspondences - *PvWAKL20* (five), and *PvWAK06*, *PvWAKL19*, *PvWAKL23*, *PvWAKL27*, and *PvWAKL33* (three each). Seven genes exhibited two correspondences, while six presented one-to-one relationships (Fig. 7B and Table S15).

Between *P. vulgaris* and *A. thaliana*, 12 syntenic pairs were identified, involving 11 *PvWAK/WAKL* and 11 *AtWAK/WAKL* genes. Although fewer in number, consistent with the closer phylogenetic relationship between *P. vulgaris* and *G. max*, these conserved collinearities indicate preservation of core *WAK/WAKL* modules across angiosperms.

Overall, 20 *PvWAK/WAKL* genes displayed conserved collinearity with *A. thaliana*, *G. max*, or both. Among them, eight genes were differentially expressed in at least one of the evaluated conditions (48 or 96 hpi) in the resistant (Ouro Vermelho) and susceptible (BRS Estilo) cultivars [4]. *PvWAKL32* and *PvWAKL33* were particularly notable. *PvWAKL32* exhibited four syntenic correspondences (two in Arabidopsis and two in soybean), and *PvWAKL33* showed five (two in *Arabidopsis* and three in soybean). All corresponding loci encoded WAK/WAKL-type kinases, typically associated with environmental perception and stress signaling.

Interestingly, these genes displayed contrasting transcriptional patterns. *PvWAKL32* exhibited a profile consistent with susceptibility at 48 hpi and resistance at 96 hpi, whereas *PvWAKL33* showed the opposite trend, being up-regulated in the resistant cultivar at 48 hpi and down-regulated at 96 hpi. Considering their conserved collinearity and opposing regulation patterns across the biotrophic (48 hpi) and necrotrophic (96 hpi) phases of infection [35, 73], *PvWAKL32* and *PvWAKL33* represent promising candidates for functional investigation, particularly in the context of WAK/WAKL-mediated signaling, developmental regulation, and environmental responsiveness in *P. vulgaris*.

To further expand the comparative context, BLASTp analyses showed that PvWAK/WAKL proteins share between 40% and 66% identity with experimentally characterized WAK/WAKL proteins from model and cultivated species (Fig. S5), including AtWAKL10 and AtWAKL22 (*A. thaliana*), BvWAK1 and BvWAK11 (*B. vulgaris*), CaWAKL20 (*C. annuum*), JrWAK12 (*J. regia*), NbWAK12 and NbWAK14 (*N. benthamiana*), SlWAKL6 (*S. lycopersicum*), TaWAKL4 (*T. aestivum*), and ZmWAK52 and ZmWAK-RLK1 (*Z. mays*) [7, 31, 32, 41, 65, 68, 74, 75]. These similarity patterns, combined with the phylogenetic relationships inferred from the expanded dataset of 113 WAK/WAKL proteins (Fig. S6), further support conserved structural and functional relationships among these groups.

### Expression patterns of *PvWAK/WAKL* genes

Differential gene expression analysis of the *PvWAK/WAKL* genes in the anthracnose-resistant (Ouro Vermelho) and susceptible (BRS Estilo) cultivars of *Phaseolus vulgaris* revealed contrasting transcriptional patterns across infection stages following inoculation with *Colletotrichum lindemuthianum* race 65 (isolate Lv134) [35]. The selected time points (0, 48, and 96 hpi) correspond to key infection phases previously characterized for this pathosystem [35, 73]: 0 hpi represents uninoculated control leaves; 48 hpi corresponds to the biotrophic phase involving appressoria, infection vesicles, and primary hyphae; and 96 hpi marks the necrotrophic phase, characterized by secondary hyphae proliferation and visible necrotic lesions in the susceptible cultivar. Six comparisons were evaluated and grouped into two temporal stages: (i) OV^48hpi^ x E^48hpi^; OV^48hpi^ x OV^0hpi^; E^48hpi^ x E^0hpi^; and (ii) OV^96hpi^ x E^96hpi^; OV^96hpi^ x OV^48hpi^; E^96hpi^ x E^48hpi^ [35]. Comparisons between 0 and 96 hpi were excluded because they merge distinct infection phases, resulting in elevated biological dispersion and reduced interpretability. Although only two biological replicates were available per condition, PCA and hierarchical clustering analyses [35] confirmed low within-group variation and clear separation among treatments, supporting the robustness of the differential expression results.

**Fig. 8:**
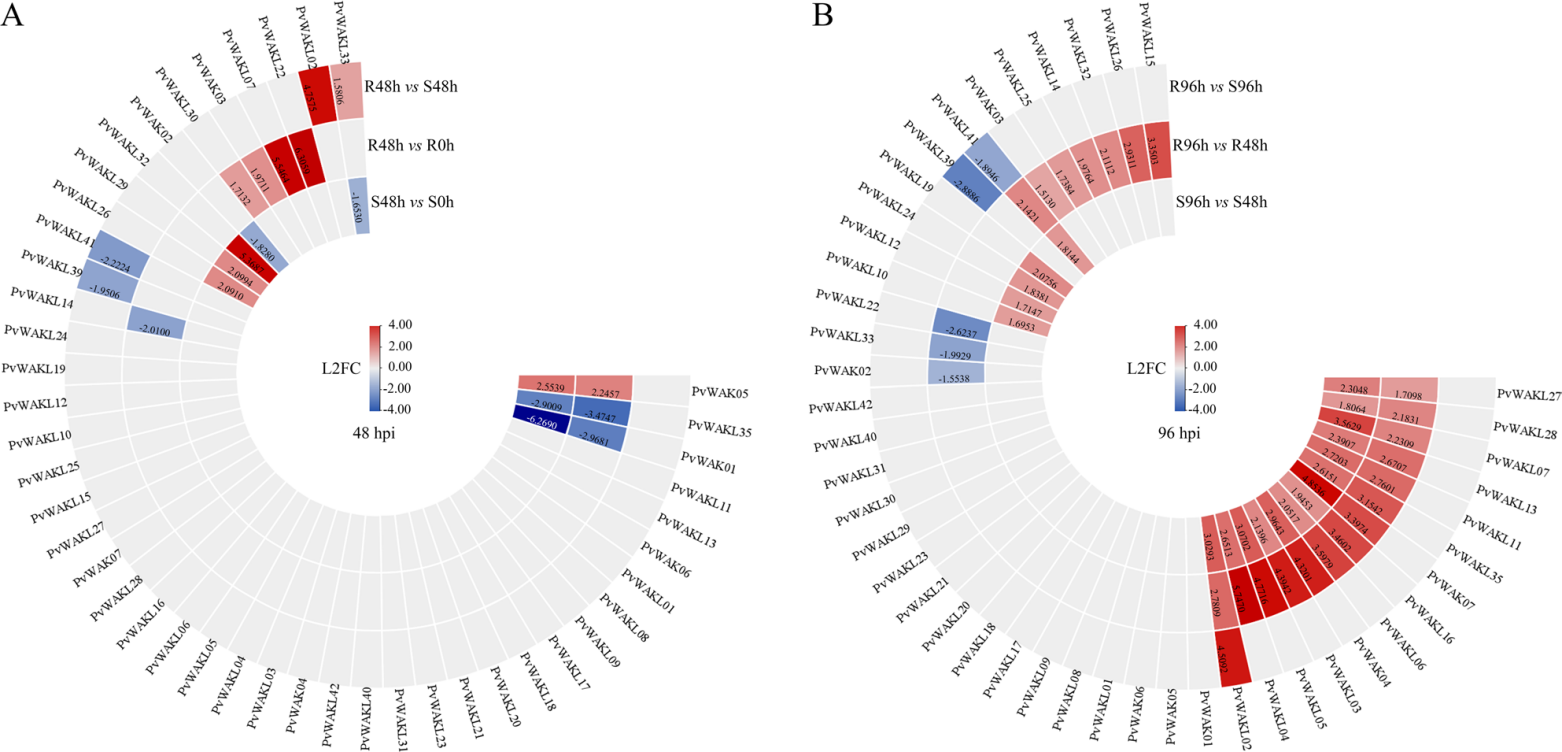
Differential expression of *PvWAK/WAKL* genes at 48 and 96 hours post-inoculation. Circular heatmaps showing log₂ fold change (L2FC) values for differentially expressed *Phaseolus vulgaris* *WAK/WAKL* (*PvWAK/WAKL*) genes in the resistant cultivar Ouro vermelho and the susceptible cultivar BRS Estilo at two post-inoculation time points. Panel **A** shows expression changes at 48 hours post-inoculation (48 hpi) and panel **B** at 96 hpi. Tiles are colored by L2FC (red = upregulated; blue = downregulated), with color intensity proportional to the magnitude of fold change. Grey cells with no numeric values correspond to genes that were not differentially expressed (Non-DEGs) under the respective condition

At 48 hpi, 29 of the 49 *PvWAK/WAKL* genes were not differentially expressed (DEGs), and three showed shared transcriptional behavior in both genotypes (Fig. 8 A). Among the DEGs potentially associated with resistance, *PvWAKL33* was notably upregulated in the resistant cultivar and downregulated in the susceptible one. Other genes upregulated in Ouro Vermelho included *PvWAKL02*, *PvWAKL22*, *PvWAKL07*, *PvWAKL03*, and *PvWAKL30*, while *PvWAK02* was downregulated in the susceptible cultivar. In contrast, genes such as *PvWAKL32*, *PvWAKL29*, and *PvWAKL26* were upregulated in the susceptible cultivar, whereas *PvWAKL41*, *PvWAKL39*, and *PvWAKL14* were downregulated in the resistant one, suggesting possible contributions to susceptibility.

At 96 hpi, 16 genes remained non-differentially expressed, while 14 were upregulated in both cultivars, indicating a general response unrelated to genotype-specific resistance (Fig. 8B). Among the DEGs with putative roles in resistance, *PvWAKL15*, *PvWAKL26*, *PvWAKL32*, *PvWAKL14*, *PvWAKL25*, and *PvWAK03* were upregulated in the resistant cultivar. Conversely, *PvWAKL41*, *PvWAKL39*, *PvWAKL22*, *PvWAKL33*, and *PvWAK02* were downregulated in Ouro Vermelho, whereas *PvWAKL19*, *PvWAKL24*, *PvWAKL12*, and *PvWAKL10* were upregulated in BRS Estilo, consistent with susceptibility-associated expression patterns (Table S16).

Together, these transcriptional profiles highlight the divergent roles of *PvWAK/WAKL* genes under compatible and incompatible interactions with *C. lindemuthianum*. Integrated with the structural, phylogenetic, and syntenic analyses presented in this study, these findings identify promising candidate genes, particularly *PvWAKL32* and *PvWAKL33*, for future functional studies aimed at elucidating the molecular mechanisms underlying anthracnose resistance and susceptibility in common bean.

## Discussion

Receptor-like kinases (RLKs) play a pivotal role in plant evolution, enabling the perception and transduction of a wide array of environmental cues [70, 76, 77]. Among them, wall-associated kinases and kinase-like proteins (WAK/WAKLs) stand out for their multifaceted roles in key biological processes, including cell elongation and expansion [78], vegetative development [7, 15, 16, 23], and hormone signaling [19, 27, 28]. These proteins are also responsive to abiotic challenges, such as heavy metal toxicity [25, 26], salinity [8], cold [13], and heat stress [14, 24]. In addition, WAK/WAKLs contribute significantly to plant immunity by mediating responses to pathogens including viruses, bacteria, nematodes, and fungi [29, 31, 64, 79–81].

Despite their biological and agronomic relevance, a comprehensive genome-wide characterization of the *WAK/WAKL* gene family in *Phaseolus vulgaris*, a species of paramount importance for global food security, had not yet been conducted. This study addresses this gap by providing the first in-depth structural, evolutionary, and transcriptional analysis of *PvWAK/WAKL* genes, with important implications for understanding their functional diversification and potential roles in anthracnose resistance.

In this study, we identified 49 *WAK/WAKL* genes in *Phaseolus vulgaris*, comprising 7 *PvWAKs* and 42 *PvWAKLs*. This number is comparable to that reported for *Cannabis sativa*, *Gossypium arboreum*, *Medicago truncatula*, and *Zea mays* [66, 68, 71]. In contrast, lower or higher counts have been observed in species such as *Juglans mandshurica* (14), *Solanum tuberosum* (29), *Hordeum vulgare* (91), *Glycine max* (94), and *Brassica rapa* (96) [33, 34, 65, 70]. These differences likely reflect species-specific gene expansion events during evolution [8]. It is important to note, however, that such comparisons must be interpreted with caution, as variations in genome quality, annotation pipelines, and domain search criteria can significantly influence gene family estimates [17].

Based on the phylogenetic classification proposed for *Arabidopsis thaliana*, *Nicotiana benthamiana*, *Solanum lycopersicum*, and *Hordeum vulgare* [31, 34], the *PvWAK/WAKL* genes were assigned to five major clades. The majority of PvWAKLs were grouped within clade III, consistent with findings in *Glycine max* [69], suggesting possible lineage-specific expansions or neofunctionalization events in closely related species. No *PvWAK/WAKL* gene was classified into clade V, which may reflect the inclusion criteria adopted in this study - namely, the presence of signal peptide (SP), GUB_WAK_bind (GWB), EGF_CA, transmembrane (TM), and serine/threonine kinase (STK) domains for *PvWAKs*; and SP, EGF_CA and/or GWB, TM, and STK domains for PvWAKLs [17, 41]. In this context, the conservation of the intracellular kinase domain across clades supports its established role in downstream signal transduction via phosphorylation [71, 82], whereas the extracellular GUB_WAK_bind and EGF domains, whose presence varies among clades, are primarily responsible for the perception of external stimuli [21, 71, 83]. This domain organization aligns with the phylogenetic structuring observed in our results, suggesting that variation in extracellular regions may have driven clade-specific diversification and functional differentiation within the PvWAK/WAKL family.

All PvWAK/WAKLs displayed predicted transmembrane domains. Experimental evidence from the literature supports plasma membrane localization of WAK proteins, such as MtWAK24 [30], and NtWAK11, NtWAK32, and NtWAK41, which also exhibit confirmed kinase activity [84]. These findings strengthen the hypothesis that PvWAK/WAKL proteins may function analogously, acting as cell surface receptors involved in signaling processes.

To better characterize the PvWAK/WAKL proteins, several biochemical and structural properties were analyzed. Variations in protein length and molecular weight reflect differences in domain composition across this gene family, particularly in the extracellular region, where EGF and/or GUB_WAK_bind domains are found. The predominance of hydrophilic profiles [68], along with the frequent occurrence of leucine and serine residues, supports the dual role of PvWAK/WAKL proteins as extracellular sensors and as mediators of intracellular signal transduction via phosphorylation. Their structural diversity suggests that PvWAKLs, in particular, may exhibit greater flexibility in recognizing and transducing environmental cues across the plasma membrane.

To provide comparative functional context, PvWAK/WAKL proteins exhibited sequence similarity around 40–66% to well-characterized WAK/WAKL members from other species, such as AtWAKL10 and AtWAKL22 (*Arabidopsis thaliana*), CaWAKL20 (*Capsicum annuum*), SlWAKL6 (*Solanum lycopersicum*), and TaWAKL4 (*Triticum aestivum*) (Figure S5). These proteins have been implicated in responses to leaf senescence, heat stress, mechanical wounding, and infections by *Fusarium oxysporum* and *Zymoseptoria tritici* [7, 16, 24, 41, 75]. The observed sequence similarity suggests that PvWAK/WAKLs may perform analogous regulatory or stress-responsive functions in *P. vulgaris*, although experimental validation and further functional analyses will be required to confirm these roles.

Although purifying selection has been reported as the predominant evolutionary force acting on this gene family in several species [17, 84], signals of positive selection have also been detected in specific regions, notably within GUB_WAK_bind domains [72], indicating potential functional adaptation. Together, these findings illustrate an evolutionary pattern characterized by cytoplasmic domain conservation alongside diversification and specialization of extracellular regions.

The gene architecture of *PvWAK/WAKL* revealed a predominance of structures with 2–3 introns, consistent with previous studies and potentially associated with faster transcriptional activation [30, 41, 66, 85, 86]. The structural conservation observed among genes within the same clade, frequently associated with gene duplication events, further supports this hypothesis [8, 17, 71, 87]. Additionally, the uneven chromosomal distribution of these genes suggests that evolutionary diversification processes, such as exon gain or loss, may have shaped the *PvWAK/WAKL* family over time [33].

To explore preliminary associations between *PvWAK/WAKL* genes and specific environmental responses, we analyzed cis-regulatory elements (CREs) within their promoter regions. In addition to core transcriptional elements (TATA-box and CAAT-box), clade-specific enrichments of regulatory categories were detected. For instance, ASR-related CREs were particularly abundant in clade IIb, BSR elements in clades III, IV, and V, LR elements in clade IIc, and DR and HR elements in clade V, highlighting possible regulatory specialization among clades. Broadly represented CREs such as MYB, MYC, ARE, W-box, WUN-motif, AAGAA-motif, as-1, ERE, ABRE, and G-box underscore the complexity of the transcriptional regulation of these genes [7, 66, 87]. Previous studies have also emphasized the role of hormone-responsive CREs in the regulation of *WAK/WAKL* genes [19].

Notably, *PvWAKL33* is positioned within a conserved syntenic block containing *WAK/WAKL* homologs with known stress-related functions in other plant species. Its Arabidopsis counterparts, *AT5G02070* and *AT3G53840*, are responsive to hormonal and environmental cues. *AT5G02070* is negatively regulated by ABA and JA and has been implicated in disease resistance in wheat through its correspondence with the candidate gene underlying the QTL QKb.cim-5BS1 for Karnal bunt resistance [88, 89]. Homologs of *AT5G02070* in *Brassica napus* are upregulated in resistant lines during *Sclerotinia sclerotiorum* infection and display opposite transcriptional responses between shoots and roots under multiple nutrient-deficiency stresses [90, 91], suggesting a context-dependent regulatory role. The second Arabidopsis homolog, *AT3G53840*, is transcriptionally responsive to gravitropic stimulation [88], consistent with the frequent involvement of WAKL-type RLKs in cell-wall-associated environmental perception.

In soybean, the syntenic genes *GmWAK23* (*Glyma.07G136900*) and *GmWAK38* (*Glyma.11G223200*) form a segmentally duplicated pair, and *GmWAK38* is strongly expressed in roots and rapidly modulated under multiple abiotic stresses (cold, heat, drought, salinity, aluminum, cadmium), indicating a central role in stress adaptation [69]. Together, these functional features across different lineages emphasize the biological relevance of the conserved genomic context surrounding PvWAKL33 and support its putative role in early defense responses, consistent with its induction in the resistant cultivar and repression in the susceptible one at 48 hpi.

Transcriptomic analysis revealed contrasting expression patterns among the 49 *PvWAK/WAKL* genes in *Phaseolus vulgaris* cultivars with differing resistance to anthracnose, highlighting the regulatory complexity underlying plant-pathogen interactions. In total, 16 genes were differentially expressed at 48 hpi and 29 at 96 hpi; however, such analyses, when based solely on a single cultivar can lead to misleading interpretations. For instance, a gene upregulated in the resistant cultivar following inoculation may be mistakenly considered resistance-related if it is also induced in the susceptible genotype, without playing a direct role in immunity. Accurate interpretation therefore requires comparative contrasts between genotypes, time points, and treatment conditions. It is important to note that the transcriptomic dataset used in this study was generated with two biological replicates per condition. Although this may reduce statistical power in leaf samples collected from soil-grown plants, previous quality assessments, including PCA and hierarchical clustering, showed clear separation among genotypes and infection stages, supporting the robustness of the DEG analysis [35].

Applying this rationale to the 49 genes analyzed, a wide range of expression profiles was observed, including non-responsive genes (33 at 48 hpi and 20 at 96 hpi), responsive but not resistance- or susceptibility-associated genes (3 at 48 hpi and 14 at 96 hpi), and genes differentially expressed in a genotype- and time-specific manner. This functional diversity, even within a relatively small gene family, reflects the intricate signaling network mediated by *PvWAK/WAKL*.

*PvWAK02*, *PvWAKL22*, and *PvWAKL33* were associated with resistance at 48 h post-inoculation and with susceptibility at 96 h, whereas *PvWAKL14*, *PvWAKL26*, and *PvWAKL32* showed the opposite pattern. *PvWAK03* and *PvWAKL02* were consistently linked to resistance at both time points, while *PvWAKL39* and *PvWAKL41* were consistently associated with susceptibility. As previously noted, syntenic WAK/WAKL homologs in other species, including *Arabidopsis*, *Brassica napus*, wheat and soybean, are known to be responsive to hormone signaling, pathogen infection, nutrient-deficiency stress and a range of abiotic stresses [69, 88–91]. These findings lend additional support to the functional relevance of the PvWAK/WAKL responsiveness detected here.

Phylogenetic context further supports the functional relevance of these proteins (Fig. S6). PvWAK02 and PvWAK03, both containing the W-box (a WRKY transcription factor binding site), group phylogenetically with BvWAK1 and BvWAK11 (Table S17), which have been implicated in nematode resistance in sugar beet [32]. PvWAKL22, showing WUN and WRE3, is positioned within the clade that includes NbWAK12 and NbWAK14 (whose silencing in *Nicotiana benthamiana* significantly increased Tomato yellow leaf curl virus (TYLCV) genomic DNA accumulation) [31], JrWAK12 (involved in female flower development) [65], and ZmWAK52 (highly expressed in maize grains, positively regulated by gibberellin, localized to the plasma membrane and cell wall, and linked to grain size and yield) [68]. PvWAKL26 is positioned in the same clade as TaWAKL4 and ZmWAK-RLK1. TaWAKL4 corresponds to the *Stb6* gene, which confers resistance to Septoria tritici blotch (STB) caused by *Zymoseptoria tritici* [75] while ZmWAK-RLK1 has been associated with quantitative resistance to Northern corn leaf blight (NCLB) [74].

Previous studies have demonstrated that proteins within the same phylogenetic clade often share gene structures, functional domain composition, structural motifs, and subcellular localization [7, 33, 68, 70], features also observed here. Taken together, these results suggest that the differentially expressed genes identified in this study may play key roles in common bean resistance to anthracnose. This hypothesis warrants detailed functional validation in future investigations.

## Conclusions

This study provides the first genome-wide characterization of the *PvWAK/WAKL* gene family in *Phaseolus vulgaris*, integrating structural annotation, phylogenetic relationships, cis-regulatory architecture, chromosomal organization, and expression profiles during the interaction with *Colletotrichum lindemuthianum*. The conserved intracellular kinase domains, the clade-specific diversification of extracellular modules, and the predominance of compact gene structures reflect a balance between evolutionary conservation and specialization within this receptor family.

Comparative genomics further revealed conserved syntenic blocks connecting *P. vulgaris* genes to *WAK/WAKL* homologs in *Arabidopsis thaliana* and *Glycine max*, many of which have experimentally established roles in pathogen perception, abiotic stress signaling, and developmental regulation. The observed 40–66% sequence identity to functionally characterized WAK/WAKLs from several species reinforces the likelihood of conserved molecular functions among PvWAK/WAKL members. Although functional assays are still required, the integration of structural, syntenic, and expression evidence highlights a small subset of genes that warrant deeper investigation. While *PvWAKL02* and *PvWAKL22* were among the genes upregulated at 48 hpi, *PvWAKL15* and *PvWAKL26* stood out at 96 hpi, particularly due to their higher expression levels relative to other highlighted genes. Additionally, *PvWAKL32* and *PvWAKL33* showed contrasting transcriptional trajectories across infection phases and were located within conserved syntenic regions, reinforcing their potential involvement in early signal perception or regulatory modulation during the anthracnose pathosystem.

Altogether, this work establishes a foundational resource for understanding WAK/WAKL-mediated signaling in common bean and provides a focused set of candidates for future functional and breeding-oriented studies.

## Supplementary Information

Supplementary Material 1.

Supplementary Material 2.

Supplementary Material 3.

Supplementary Material 4.

Supplementary Material 5.

Supplementary Material 6.

Supplementary Material 7.

## Data Availability

The RNA-Seq datasets analyzed in this study are publicly available in the NCBI Sequence Read Archive under the BioProject accession number [PRJNA1219552](https:/www.ncbi.nlm.nih.gov/bioproject/PRJNA1219552) . Processed expression data (log₂ fold changes) are provided in Supplementary Table S16. Phylogenetic trees, domain annotation tables, and analysis scripts are provided as supplementary material. All other supporting data are included in this published article and its supplementary information files.
