# Supplementary figures and images for: The WAK/WAKL gene family in Phaseolus vulgaris: genomic characterization and expression under Colletotrichum lindemuthianum infection
Source: BMC Genomics. 2026 Jan 24;27:210. doi: 10.1186/s12864-026-12531-2 (PMC12911021; doi:10.1186/s12864-026-12531-2)

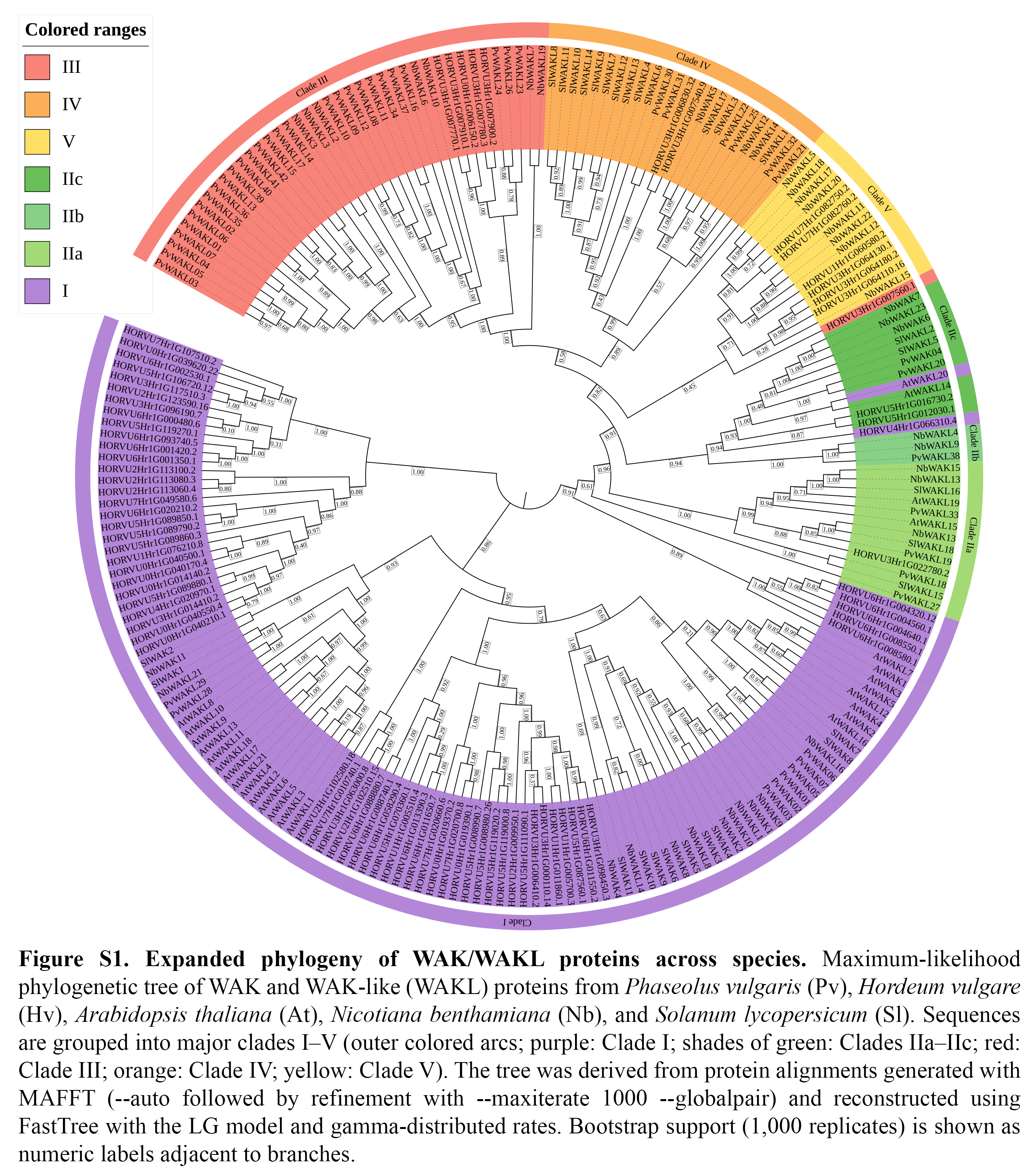

Supplement: Supplementary file 1 — Supplementary Material 1. [file 12864_2026_12531_MOESM1_ESM.png]

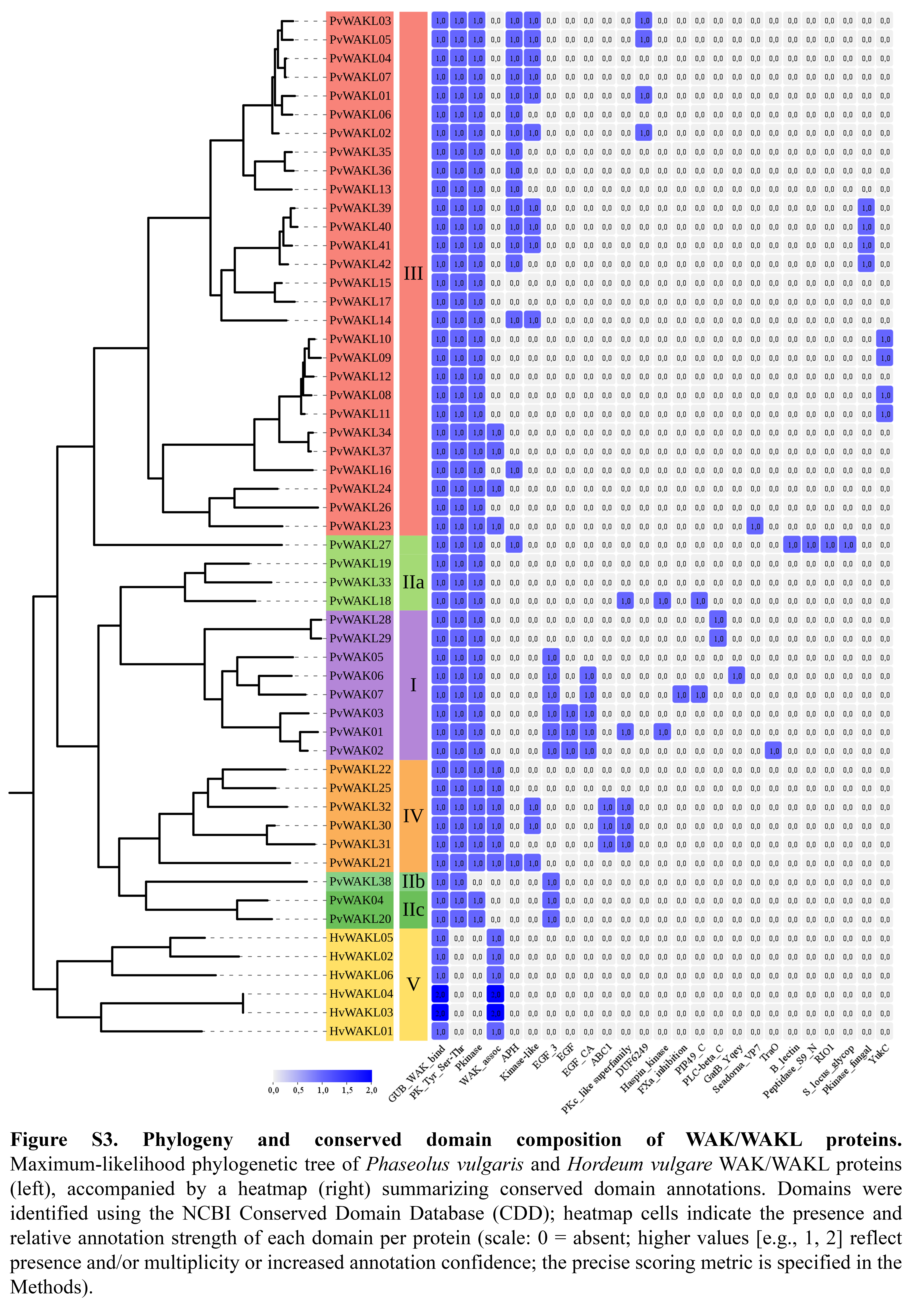

Supplement: Supplementary file 3 — Supplementary Material 3. [file 12864_2026_12531_MOESM3_ESM.png]

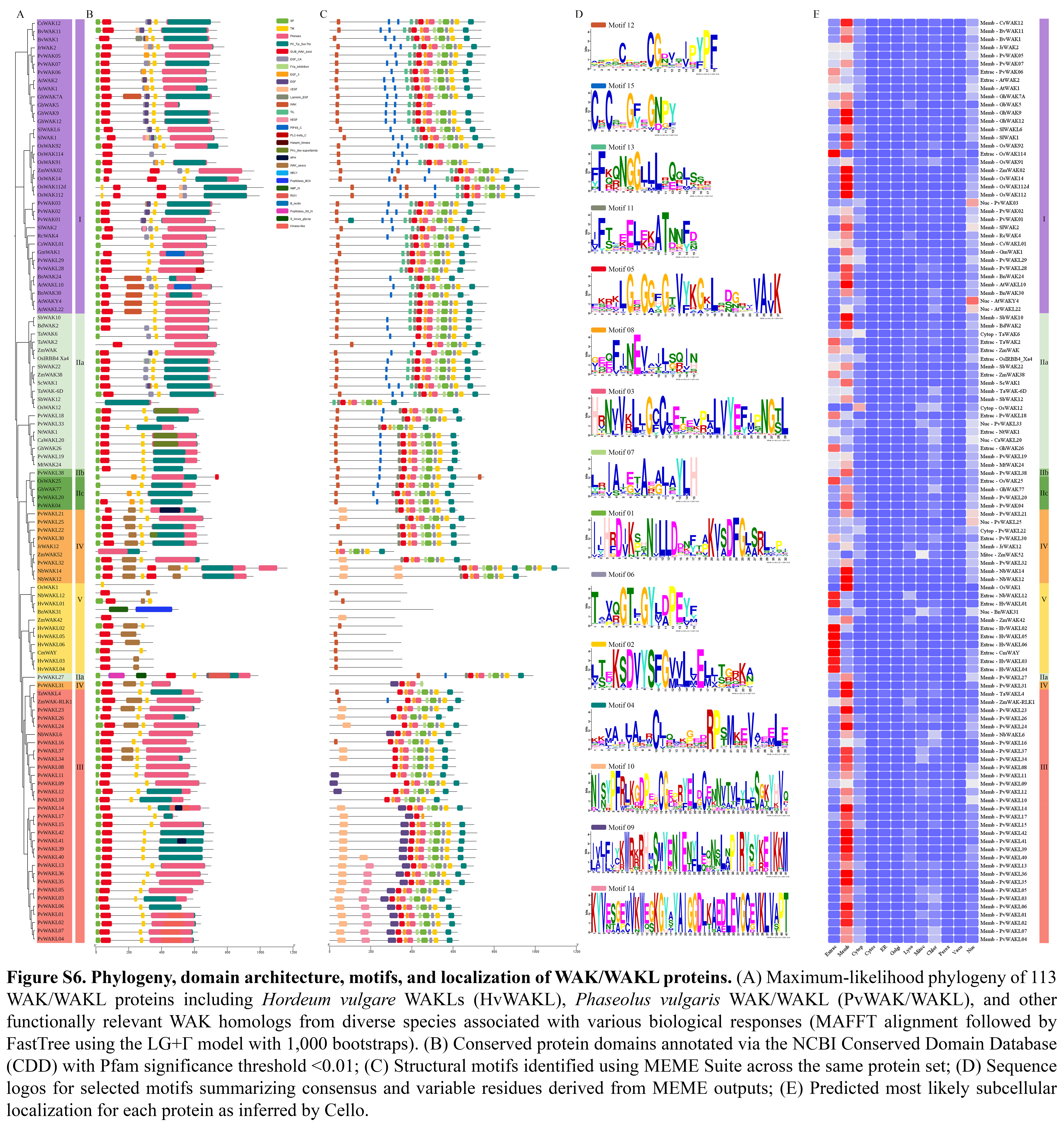

Supplement: Supplementary file 6 — Supplementary Material 6. [file 12864_2026_12531_MOESM6_ESM.png]
